# Temperate phages increase antibiotic effectiveness in a *Caenorhabditis elegans* infection model

**DOI:** 10.1128/mbio.01621-25

**Published:** 2025-08-18

**Authors:** Rabia Fatima, Gayatri Nair, Jordan Mayol, Lesley T. MacNeil, Alexander P. Hynes

**Affiliations:** 1 Department of Biochemistry and Biomedical Sciences, McMaster University 3710, Hamilton, Ontario, Canada; 2 Department of Medicine, McMaster University 152997, Hamilton, Ontario, Canada; 3 Farncombe Family Digestive Health Research Institute, McMaster University 3710, Hamilton, Ontario, Canada; 4 Michael G. DeGroote Institute for Infectious Disease Research, McMaster University 3710, Hamilton, Ontario, Canada; The University of Texas Health Science Center at Houston, Houston, Texas, USA

**Keywords:** bacteriophage therapy, *C. elegans*, phage antibiotic synergy, temperate phages, lysogeny, induction

## Abstract

The bactericidal nature of obligately lytic bacterial viruses (phages) is of increasing interest for the treatment of drug-resistant bacterial infections, often alongside antibiotics. By contrast, temperate phages are largely ignored due to their ability to lie dormant within the bacterial host. However, these phages often undergo a lytic cycle. Furthermore, in their dormant state, they switch to lytic replication in response to triggers, such as antibiotics, that stress the bacterial host. Recent reports of antibiotics synergizing with temperate phages *in vitro*, termed “temperate phage-antibiotic synergy” (tPAS), present a potentially scalable opportunity to make use of these abundant entities in therapy. Here, we employ *Caenorhabditis elegans* as a model to test the efficacy of temperate phages. *In vivo*, temperate phage Hali alone results in 60% dormancy in the bacterial survivors. However, the antibiotic ciprofloxacin can abolish dormancy of the phage—even in a ciprofloxacin-resistant *Pseudomonas aeruginosa* clinical strain. The phage Hali-ciprofloxacin pairing increased the lifespan of *P. aeruginosa*-infected worms to that of the uninfected control, at doses where neither the phage nor the antibiotic had any effect alone. Complete rescue was also observed in worms infected with a phage-carrying strain treated with the otherwise ineffective antibiotic, supporting that the phage—even in its dormant form—can greatly enhance antibiotic effectiveness. This illustrates potential “accidental” phage therapy when antibiotics are prescribed. As the first *in vivo* demonstration of tPAS, our work establishes *C. elegans* as a model for studying it, greatly expanding the therapeutic potential of temperate phages.

**IMPORTANCE:**

Bacterial viruses (phages), which can go dormant in their host, are not considered suitable for phage therapy. Building on prior work showing antibiotics synergize with these phages by preventing dormancy, we test the ability of temperate phages—in combination with antibiotics—in an animal model of infection. Not only were the combinations extremely effective at doses that would do nothing alone, even when the bacterium was resistant to the antibiotics—but we also found that whether the dormant phage pre-existed (as it does in about 75% of bacteria) or not determined the success of antibiotic treatments, suggesting many antibiotic treatments are already a form of “accidental” phage therapy.

## INTRODUCTION

With the rising antimicrobial resistance crisis, bacteriophages (phages) are increasingly explored as adjuvants or alternatives to antibiotics. Most work has focused on strictly lytic phages, given their immediate bactericidal nature (1–5). However, it is estimated that 50%–75% of all bacteria contain at least one dormant phage, termed temperate or lysogenic (6, 7). A major clinical concern specific to temperate phages comes at the level of the integrated phage (prophage), conferring immunity to its host against subsequent phage infections through a variety of mechanisms (8). This leads to a rapid regrowth of phage-generated resistant “mutants” and could readily contribute to treatment failure. However, carrying a prophage can be costly. Lysogens (bacteria-carrying prophages) of several *Pseudomonas aeruginosa* phages demonstrate defects in bacterial motility, swarming, and twitching (9). Clearly, the prophage can have a lasting influence on its host physiology, virulence, and pathogenesis.

A common change in the lysogen is that the prophage can be highly responsive to stressors of its bacterial host. In *Escherichia coli* phage Lambda lysogens*,* the phage can sense host DNA damage to switch from lysogenic to lytic replication, resulting in cell lysis (10). In the gastrointestinal tract of monoxenic mice, 1%–2% of Lambda lysogenic bacteria were lysed *per* generation due to spontaneous prophage induction (11). Hence, prophage excision itself may impose a significant fitness burden, especially in environments of high stress.

Furthermore, lysogens can exhibit increased antibiotic sensitivity, monitored as decreases in minimum inhibitory concentration (MIC), compared to the parent strain (12, 13). Temperate phages can also synergize with antibiotics (2, 12–14), increasing phage production (2) or decreasing the antibiotic MIC (12–14). When antibiotics specifically influence the phage lysis-lysogeny switch, this is referred to as temperate phage-antibiotic synergy (tPAS). Given that temperate phages are dominant within the human body (15), their existing interaction with antibiotics likely already plays a role in treatment outcomes.

Current animal models for phage therapy—wax moth larvae (16), hamsters (17), and zebrafish (18)—offer valuable insight but present several scalability concerns, with each animal requiring individual manipulation. Here, we developed the nematode *Caenorhabditis elegans* as an infection model for studying the *in vivo* efficacy of a temperate phage-antibiotic combination against the multidrug-resistant bacteria *P. aeruginosa. C. elegans* offers several key advantages: short, measurable lifespan and ability to generate large, germ-free age-synchronized populations. It is widely used to study *P. aeruginosa* pathogenesis (19) and evaluate antibacterial drug efficacy (20, 21).

Only a few studies have used *C. elegans* to investigate phage therapy, focusing on lytic phages (22–25), with only one study specifically for *P. aeruginosa* (26). None have tested antibiotics alone or in combination with their phage of interest. We explored *C. elegans* to quickly iterate and screen a large set of temperate phage-antibiotic pairings that synergized *in vitro* against *P. aeruginosa* (13).

## RESULTS AND DISCUSSION

### Validation of *C. elegans* as an infection model

To investigate whether co-administration of temperate phage and antibiotic can result in bacterial killing in an *in vivo C. elegans* model, we first validated *P. aeruginosa* infection with a clinical strain of interest, C0400. C0400 is a ciprofloxacin-resistant strain where co-administration of temperate phage Hali and ciprofloxacin was able to re-sensitize the strain to the antibiotic *in vitro* (13). For our experiments, we employed worms carrying a temperature-sensitive mutation (*bn2*) in the germ-line proliferation regulator *glp-4*. Mutant animals maintained at the restrictive temperature of 25°C are sterile, which bypasses the need to separate adults from their offspring in survival assays (27). *P. aeruginosa* C0400 can colonize the intestine at levels comparable to the strain PA14, commonly used in *C. elegans* for studying *P. aeruginosa* pathogenesis (27), and higher than the *E. coli* OP50 control (Fig. S1a). Similarly, *P. aeruginosa*-fed worms begin dying after 3 days, much faster than the OP50 control, with complete population death observed by day 10 (Fig. S1b). The lifespan data closely match previously reported survival data of the *glp-4(bn2*) strain exposed to OP50 and PA14 (27), validating that the clinical *P. aeruginosa* strain C0400 is suitable for investigating tPAS in this *in vivo* model.

### Ciprofloxacin selects against lysogens *in vivo*

**Fig 1:**
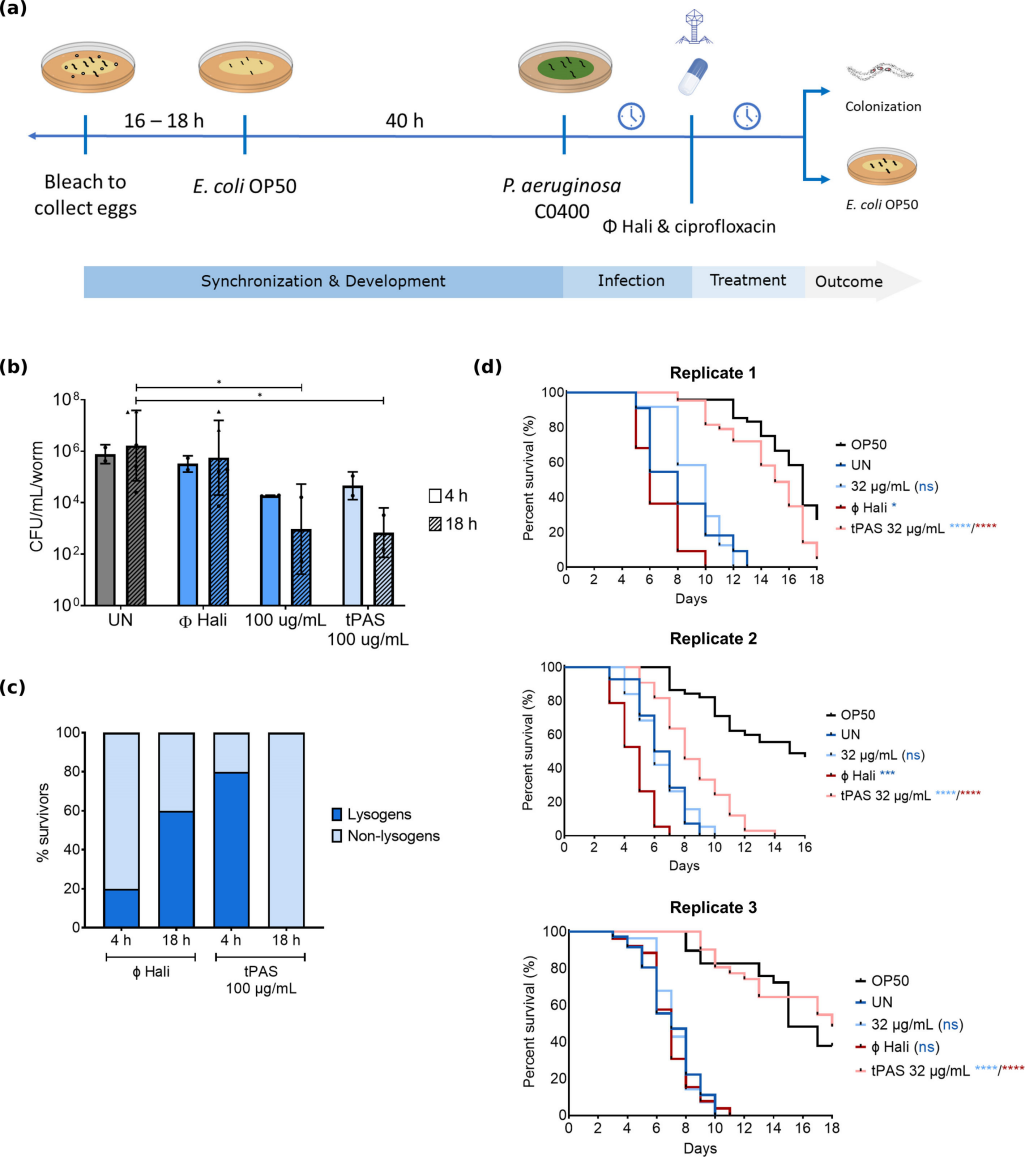
Phage Hali and ciprofloxacin select against lysogen survivors and can increase survival of *P. aeruginosa* infected worms on a standard *E. coli* OP50 diet after treatment. (**a**) Illustrative representation of the assay used for testing the efficacy of temperate PAS in a *C. elegans* model of *P. aeruginosa* infection. Worms are bleached to collect eggs, which are then hatched and synchronized to L1 stage. Animals are developed on *E. coli* OP50 and infected with *P. aeruginosa,* followed by phage and/or antibiotic treatment. The outcome of treatment is monitored by bacterial colonization and lifespan on a regular bacterial diet. Each assay was done in independent biological replicates. (**b**) *C. elegans* intestinal colonization measured as CFU/mL/worm after 48 h C0400 infection, followed by 4 h or 18 h of treatment with phage Hali (1 × 10^9^ plaque forming units or PFU) and/or ciprofloxacin. Data are shown as mean ± SD, each data point is a biological replicate plated in technical triplicate, except ones denoted by triangle, which are single technical replicate, with ~50–100 worms per condition. The two treatment durations were tested in independent trials. Values were compared using two-way ANOVA with a multiple comparison Tukey test post hoc with conditions only compared within their treatment duration, *P-value 0.01–0.05. (**c**) Frequency of lysogeny within survivors of phage Hali alone and combined with ciprofloxacin (tPAS). Twenty survivors from a single biological replicate were tested for each condition. (**d**) Life span of worms on OP50 after 4 h *P*. *aeruginosa* infection, followed by 18 h of phage Hali and/or ciprofloxacin treatment. Approximately 50 worms were added to each treatment condition, tested in three independent biological replicates performed on separate days. Survival curves were compared using the log-rank (Mantel-Cox) test with one pair compared at a time, ns = not significant, *P ≤ 0.05, ***P ≤ 0.001, and ****P ≤ 0.0001. Phage and antibiotic alone were each compared to the untreated, and tPAS was compared to both the phage alone and antibiotic alone, denoted by the color of the significance value.

To test whether tPAS reduces *P. aeruginosa* loads *in vivo*, we tested 4 h and 18 h (Fig. 1a) treatment with ciprofloxacin (0.25–180 µg/mL), alone or combined with phage Hali. There was no effect at both higher and lower antibiotic doses for the 4 h treatment (Fig. S2a and b) and a high degree of biological variability between replicates at lower concentrations after 18 h (Fig. S2c and d). There was no reduction in colonization when treated with phage alone (Fig. 1b). There was a 1–2 log_10_ reduction observed with 100 µg/mL ciprofloxacin but no synergistic reduction for tPAS for either treatment duration.

Despite no reduction in bacterial loads, tPAS resulted in primarily lysogen population within 4 h treatment (80% compared to 20% for phage alone) and a reduction in frequency of lysogeny to zero with longer treatment duration (Fig. 1c). Compared to phage alone, tPAS selectively biases the phage away from lysogeny *in vivo,* preventing the regrowth of lysogens that would otherwise form 60% of the population. This initial formation, followed by depletion of lysogens, is consistent with the previously reported induction-based mechanism of this interaction (13). While all treatments modestly reduced bacterial load, the combination drastically altered the nature of those bacteria, selecting against lysogens. Given these findings, we sought to determine whether this bias was sufficient to improve worm survival.

### Combined temperate phage and ciprofloxacin rescue *P. aeruginosa*-infected worms

First, we carried out the same assay but moved the animals to their standard *E. coli* OP50 diet after 18 h of treatment to measure lifespan. We performed the lifespan assay with a shorter 4 h of infection as we did not observe any rescue from even the antibiotic at high doses with a longer infection duration, suggesting that worms were moribund (data not shown). With this shorter infection, the worms died within maximum 13 days, much earlier than the uninfected OP50 control (Fig. 1d). Phage Hali was not only ineffective, but it also showed a worse survival outcome in two of the three trials (Fig. 1d). Ciprofloxacin alone at low dose (2 µg/mL) did not reliably rescue survival alone or combined with phage (Fig. S3a) and at much higher antibiotic dose (92 µg/mL), the antibiotic alone was sufficient for rescue (Fig. S3b).

Ciprofloxacin treatment alone at 32 µg/mL, which is lower than the effective dose, did not increase survival (Fig. 1d). In comparison, tPAS had a marked improvement in lifespan to levels comparable to the uninfected OP50 control in all trials. The *in vitro* work that had shown synergy between phage Hali and ciprofloxacin (13) translated to a robust, reproducible *in vivo* increase in worm survival when neither agent alone had any detectable effect.

We then asked whether survival was driven by bacterial load, performing the colonization assays as carried out earlier (Fig. 1b) but found that treatment outcomes for these shorter 4 h infections did not correlate with bacterial loads (Fig. S4). It is possible that a small number of bacteria persist below the limit of detection for ciprofloxacin alone and may later regrow, hence show no rescue. Alternatively, either the phage-antibiotic combination is benefiting the worms through faster eradication, qualitatively different endpoints for the bacterium (phage-mediated lysis vs antibiotic-mediated death), or the presence of transient lysogeny mitigates the virulence of the strain. 

### Lysogen-infected worms can be rescued with an antibiotic

**Fig 2:**
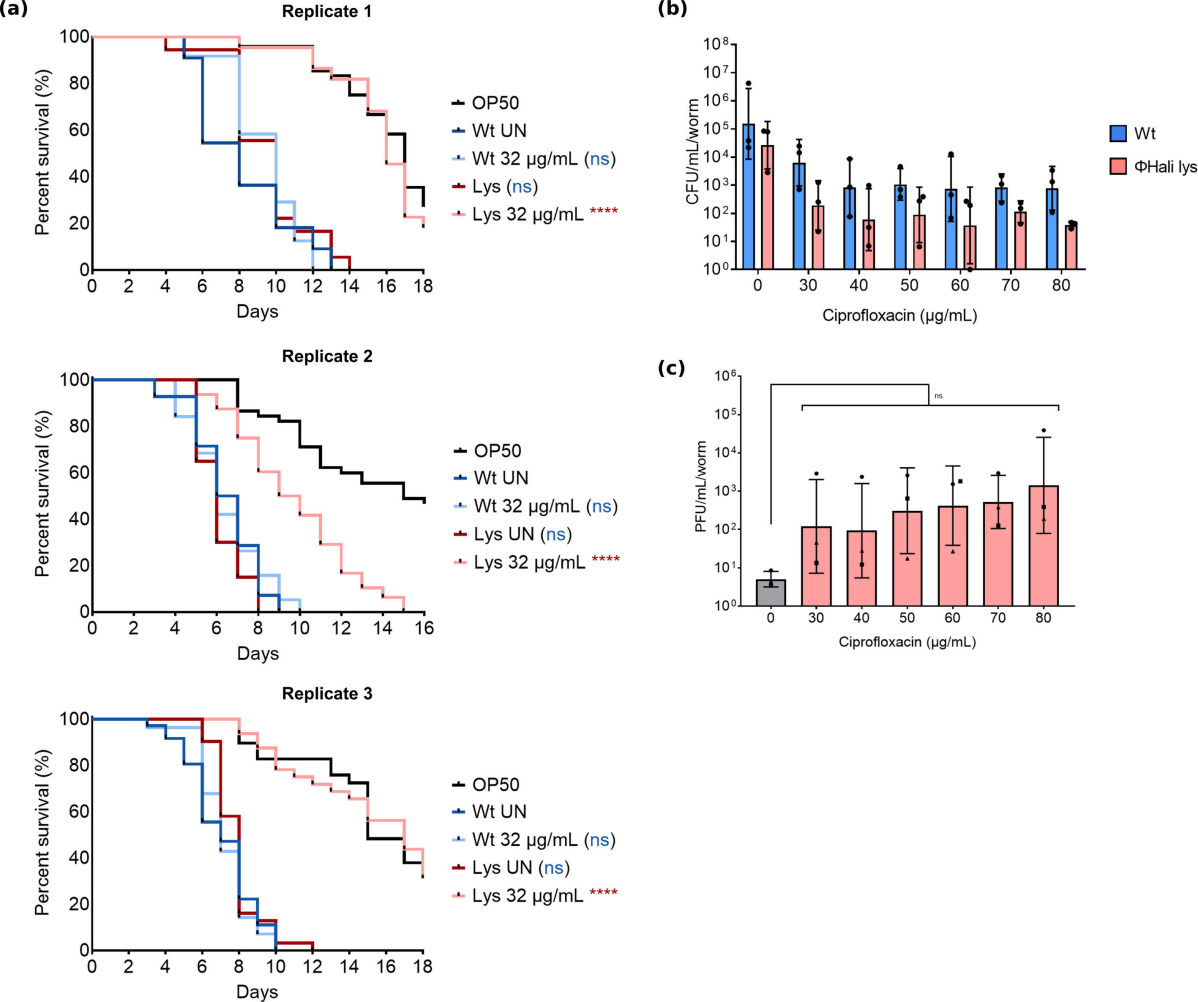
Phage Hali lysogen-infected worms can be rescued with antibiotic alone, but the lysogen is not more sensitive to the antibiotic. (**a**) Life span of worms on OP50 after 4 h *P*. *aeruginosa* C0400 wild-type and phage Hali lysogen infection, followed by 18 h of ciprofloxacin treatment. Approximately 50 worms were added to each condition at the time of treatment, tested in three independent biological replicates performed on separate days. Survival curves were compared using the log-rank (Mantel-Cox) test with one pair compared at a time, ns = not significant, *P ≤ 0.05, ***P ≤ 0.001, and ****P ≤ 0.0001. Lysogen untreated was compared to wild type untreated, and antibiotic treatment was compared to its respective no antibiotic control, as denoted by the color of the significance value. (**b**) *C. elegans* intestinal C0400 wild type and phage Hali lysogen colonization measured as CFU/mL/worm after 48 h infection, followed by 18 h of treatment with ciprofloxacin. Bacterial load was compared using two-way ANOVA with a Bonferroni test. All comparisons were not significant. (**c**) Corresponding phage quantification shown as PFU/mL/worm for the lysogen-infected worms treated with ciprofloxacin. Phage titer was compared using a one-way ANOVA with a Dunnett test; ns represents not significant. All bar graph data are shown as mean ± SD where each data point is a biological replicate carried out with roughly 100 worms per condition, plated in technical triplicates, except for ones denoted by circle and square in (**c**), which were plated in single replicates.

To determine whether the formed lysogens were differentially virulent, we infected worms with wild-type C0400 and a C0400 phage Hali lysogen, followed by ciprofloxacin treatment and subsequently monitored survival after treatment. Untreated, the pathogenicity of the lysogen and wild-type strains did not differ (Fig. 2a). Intriguingly, lysogen-infected worms could be completely rescued with 32 µg/mL ciprofloxacin, which was insufficient to rescue worms infected with the wild-type C0400. This strongly supports the notion that the tPAS observed is primarily driven by the interaction between the prophage and the antibiotic, as any phages induced (spontaneously or by the antibiotic) would have little effect on the lysogens, which are immune to superinfection.

This finding is mechanistically important because one explanation for tPAS is that phage administration modestly reduces bacterial load before lysogen regrowth, making bacteria more sensitive to the antibiotic—known as density-dependent antibiotic sensitivity (28). However, here the phage and antibiotic are truly synergistically interacting. This similar rescue observed to tPAS (Fig. 1d) also means that whether the phage is co-administered or present as a prophage, it can enhance antibiotic effectiveness.

Considering that lysogens are often more sensitive to environmental stressors because of prophages that are responsive to these triggers (12, 13, 29), we assessed whether carrying a prophage burdens the bacteria *in vivo*, especially in the presence of antibiotics, by measuring colonization of worms infected with wild-type or lysogen and treated with ciprofloxacin. Here, we also once again observed a high degree of biological variability in colonization post-treatment with no observed difference between wild-type and lysogen colonization post-ciprofloxacin treatment (Fig. 2b).

In colonization assays, we could often see eradication without rescue, and in our rescue assays, we could see rescue without a difference in colonization. This reinforced the idea that bacterial loads at the times selected are not a good predictor of treatment efficacy, but the kinetics or manner of their death can impact survival. While not statistically significant, lysogen-infected worms had 10-fold to 100-fold higher phage counts after antibiotic treatment, consistent with induction (Fig. 2c). Given the trend toward reduced bacterial loads (Fig. 2b), this likely underestimates induction magnitude. This is consistent with an *in vitro* report showing phage Hali lysogens have four-fold lower ciprofloxacin MIC compared to wild type due to prophage induction (13).

While the increase in worm survival did not correlate with a reduction in bacterial load, the increased survival and phage counts of phage Hali lysogen-infected worms treated with ciprofloxacin suggest that carrying an inducible phage could impose a major fitness cost under mild antibiotic stress.

Given the abundance of prophages within bacterial hosts (6, 7) and that pathogens are well-known reservoirs of prophages (30), this effect is likely already taking place during antibiotic treatments, in a form of “accidental phage therapy.” It is important to note that this is likely phage dependent, not just with regard to its integration site and receptor, but especially in its ability to respond to antibiotic triggers. The *P. aeruginosa* LES strain common in cystic fibrosis patients is known to contain several prophages that can be induced with ciprofloxacin *in vitro* (30). In addition, from patient data, the induction of these phages was shown to play a crucial role in the regulation of the pathogen in CF lungs (31). However, it is challenging to correlate the effects of long-term antibiotic prescription to pathogen load and phage induction since the data are incomplete. James et al. (31) do propose that treatments that result in phage induction could be promising approaches to tackling *P. aeruginosa* load in these patients, not without caution in case of phage-encoded virulence factors (31).

In conclusion, temperate phages are avoided in therapy due to their ability to lysogenize and potentially facilitate the transfer of antimicrobial resistance and virulence genes. However, temperate phages are not inherently more likely to mediate such gene transfer than strictly lytic phages (32). They have an enormous untapped potential due to their ability to respond to environmental stress, such as antibiotics, to switch into lytic replication, killing their host in the process. Here, we show that *C. elegans* is a suitable model for studying the *in vivo* efficacy of tPAS, but this can easily be extended to traditional PAS with virulent phages. While we did not observe synergistic reduction in bacterial loads, ciprofloxacin strongly selects against phage Hali lysogens. While phage Hali is completely insufficient to rescue the worms, phage and ciprofloxacin combined increased the lifespan of infected worms at an otherwise ineffective antibiotic dose. Complete rescue can be seen for the lysogen-infected worms treated with the antibiotic. The phage, even in its prophage form, greatly enhanced antibiotic effectiveness. This suggests to us that this kind of “accidental” phage therapy already often occurs when antibiotics are prescribed.

## MATERIALS AND METHODS

### Bacterial strains and growth conditions

*P. aeruginosa* strain PA14 was obtained from Dr. Lori Burrows, McMaster University, and *P. aeruginosa* C0400 was from the McMaster IIDR Wright clinical isolate collection. Bacterial strains were grown in 10 mL of lysogeny broth (LB) at 37°C with 250 rpm shaking (Ecotron, Infors HT, Quebec, Canada). Overnight culture was diluted 1:100 in LB broth and grown with shaking to O.D. 0.2 to prepare same day cultures. For growth on solid media, 1% (wt/vol) of LB agar and 0.75% (wt/vol) of LB soft agar were used. All plates were incubated at 37°C for overnight growth.

To prepare *C. elegans* plates, bacterial strains were streaked out on 1% LB agar plates and incubated overnight. A single colony was inoculated into 10 mL LB broth, followed by overnight incubation with shaking. To seed plates, 200 µL of overnight culture was used for 100 mm plates and 100 µL was used for 60 mm plates. The plates were incubated overnight at 37°C prior to transferring worms on them.

### Phage propagation and titration

Phage Hali, infecting *P. aeruginosa* PA14 and C0400, was used in this study. Phage lysates were prepared either using a primary or secondary amplification. Briefly, primary amplification was performed by inoculating 10 mL LB broth with bacteria and phage from frozen glycerol stocks for amplification to take place overnight. Secondary amplification was performed as needed to increase phage titer by inoculating 10 mL of the same-day culture prepared in LB with 50 µL of primary amplification. Phage amplifications were filtered using a 0.45 µm filter. Phages were quantified by standard spot test or full plate plaque assay.

### *C. elegans* strains and growth conditions

All experiments were performed using *C. elegans* SS104, a temperature-sensitive sterile strain, *glp-4* (*bn2*) available from the *Caenorhabditis* Genetic Center (CGC, https://cgc.umn.edu/). Worms were maintained at 15°C on 100 mm nematode growth media (NGM) plates (33) seeded with overnight cultures of *E. coli* OP50, prepared as indicated above. Eggs were collected by hypochlorite treatment (34). Eggs were rocked approximately 18 h at 20°C to synchronize to the L1 larval stage. L1 worms were plated on 100 mm NGM plates seeded with *E. coli* OP50 and developed to young adults at 25°C, a restrictive temperature at which they are sterile, for approximately 40 h.

### Antibiotic stock preparation

Ciprofloxacin (hydrochloride) was obtained from Cayman Chemicals (Catalog 14286-5, Ann Arbor, Michigan, USA). Working stocks were prepared in nuclease-free water. The minimum inhibitory concentration of each newly prepared batch was re-evaluated prior to use as reported previously (13).

### *C. elegans P. aeruginosa* infection

For *P. aeruginosa* infection, young adults were washed off NGM OP50 plates with 10 mL M9 (1.5 g KH_2_PO_4_, 3 g Na_2_HPO_4_, 2.5 g NaCl for 500 mL, autoclave, then final 1 mM MgSO_4_) and washed three times to remove excess bacteria. During each wash, they were pelleted by centrifugation at 1,200 rpm for 2 mins. Worms were transferred to 100 mm Slow Killing Media plates (SKM; 0.35% peptone, 50 mM NaCl, 2% agar, 1 mM CaCl_2_, 5 µg/mL cholesterol, 1 mM MgSO_4_, 20 mM KH_2_PO_4_, 5 mM K_2_HPO_4_) seeded with different *P. aeruginosa* strains for appropriate infection duration as indicated in the figure legends. Worms were also plated on *E. coli* OP50 for the life span assay as a control.

### Preparing *P. aeruginosa*-infected worms for treatment

After infection with *P. aeruginosa,* worms were washed off SKM plates with 10 mL M9, followed by another three M9 washes. Each wash step consisted of pelleting the worm for 2 min at 1,200 rpm and removing the supernatant, except for the third wash, in which they were allowed to settle by gravity. To further dilute the bacteria, 1 mL of M9 with 10 µg/mL of tobramycin was added, and worms were pelleted by centrifugation, followed by transfer to an unseeded 100 mm 10 µg/mL SKM tobramycin plate for 45 min in the biosafety cabinet to allow the removal of surface-attached bacteria. After crawling, they were washed off SKM tobramycin plates with 10 mL M9 tobramycin and subsequently washed another three times with 10 mL M9 tobramycin and finally three times with M9 alone to dilute out the tobramycin.

### Colonization assay

To measure the efficacy of tPAS on bacterial colonization, approximately 50–100 infected worms were added to a 2 mL tube manually, along with ciprofloxacin (final concentration tested 0.5–32 µg/mL at two-fold increments, then 32–100 µg/mL in increments of 10 µg/mL) and/or phage Hali at 1 × 10^9^ PFU. The final volume was topped up to 1 mL using SKM broth. SKM alone was used as a negative control. Tubes were incubated for 18 h with 150 rpm horizontal shaking, and a colonization assay was carried out subsequently.

Following treatment, worms were settled by centrifugation for 2 min at 1,200 rpm or by gravity when working with more than 10 samples. After removing the supernatant, worms were washed three times with 1 mL M9, followed by once with 1 mL 10 µg/mL M9 tobramycin. To remove surface-attached bacteria, they were transferred to 60 mm 10 µg/mL SKM tobramycin plates for 45 min. At this point, the total number of animals for each condition was counted.

Worms were washed off plates with 2 mL 120 µM levamisole in M9 (LM buffer). The number of worms remaining on the plate was counted to determine the number of animals per condition going into the lysis step. Animals were washed with 1 mL 10 µg/mL tobramycin LM buffer, once for 4 h treatment and three times for 18 h treatment. To dilute out the tobramycin, worms were additionally washed three times with 1 mL M9. In the last wash, 500 µL of the supernatant was transferred into a new tube to quantify any residual bacteria, which will be deducted from the bacterial load calculation later.

Approximately 10 sterile silica carbide beads were added to each tube, and worms were lysed for 1 min at speed 5 using the Bead Mill 4 (Fisherbrand, catalog 15340164). Lysed fractions and the last wash supernatants were serially diluted 10-fold in LB up to 10^−7^, and 3 µL was spotted onto a 1% LB plate in technical triplicate, along with one replicate for the last wash. LB only and M9 with beads were spotted as controls. Plates were incubated at 32°C to prevent overgrowth, and colony-forming units were counted after an overnight incubation. Bacterial load was determined by subtracting the counts from the last wash, and the cfu/mL was normalized to the number of animals per condition.

### Frequency of lysogeny

Frequency of lysogeny in the survivors was determined as previously described (13). Twenty colonies for each condition were streak purified three times on 1% LB plate and inoculated in 200–250 µL LB broth overnight in a narrow 96-well plate. Wild-type culture, C0400 phage Hali lysogen, and LB broth were added as controls. The plates were incubated for 18–24 h at 37°C with no agitation. The inoculated plates were stamped on an agar overlay of wild-type C0400 using a disposable pin replicator. Plates were also stamped onto 1% LB with no agar overlay to serve as growth controls. Frequency of lysogeny was calculated as a percentage of the total number of survivors that resulted in clearing of the wild-type host.

### Survival on OP50 after treatment assay

*C. elegans* were infected with *P. aeruginosa* strains, or OP50 as a control, for 4 h and washed off as described above. The volume of infected worms per strain was adjusted to 1 worm/µL, and approximately 100 worms were sorted into each well of a 96-well plate using the COPAS FP (Union Biometrica, Holliston, MA). These 100 worms were then transferred to a 2 mL tube, along with 50 µL of 20× ciprofloxacin stock (final concentration tested 0.25–32 µg/mL at two-fold increments, then 32–100 µg/mL in increments of 10 µg/mL), and phage Hali at 1 × 10^9^ PFU. Nuclease-free water was used as a substitute for antibiotic and LB in place of phage. The final volume was topped up with SKM broth to 1 mL. The worms were incubated in the treatment for 18 h, shaking horizontally at 150 rpm.

Following treatment, animals were washed three times with 1 mL M9, allowed to settle by gravity in the third wash, and once with 1 mL 10 μg/mL M9 tobramycin. They were transferred to 60 mm SKM tobramycin plates for 45 min to crawl, after which they were washed off with 2 mL M9 tobramycin. They were washed once more with 1 mL M9 tobramycin, ending with the final three washes with M9 alone.

After the last wash, animals were moved to 60 mm SKM plates seeded with OP50. Plates were incubated at 25°C and scored daily for survival starting on day 3, with dead worms removed each day. Survival was plotted using the Kaplan–Meier method.

### Statistical tests

Details of the statistical tests can be found in the figure legend. N denotes a biological replicate performed from an independently grown culture with appropriate technical replicates indicated in the figure caption. Quantitative values are represented by mean ± SD. All statistical analysis was done using GraphPad Prism 8.0.2 or 10.3.0 (GraphPad Software, Inc., CA, US), with *P* value ≤ 0.05 considered significant.

## SUPPLEMENTAL MATERIAL

Fig. S1 mbio.01621-25-s0001.eps *P. aeruginosa* C0400 can colonize and kill *C. elegans*.

Fig. S2 mbio.01621-25-s0002.eps Longer treatment duration shows variability in bacterial colonization.

Fig. S3 mbio.01621-25-s0003.eps Ciprofloxacin alone could rescue life span of *P. aeruginosa* infected worms on standard *E. coli* OP50 diet after treatment.

Fig. S4 mbio.01621-25-s0004.eps Shorter infection prior to treatment does not provide enough resolution to measure bacterial colonization.

Legends mbio.01621-25-s0005.docx Supplemental figure legends.

Raw Data mbio.01621-25-s0006.xlsx Raw data for all figures.

ASM does not own the copyrights to Supplemental Material that may be linked to, or accessed through, an article. The authors have granted ASM a non-exclusive, world-wide license to publish the Supplemental Material files. Please contact the corresponding author directly for reuse.
