# Supplementary material for: Temperate phages increase antibiotic effectiveness in a Caenorhabditis elegans infection model
Source: mBio. 2025 Aug 18;16(9):e01621-25. doi: 10.1128/mbio.01621-25 (PMC12421866; doi:10.1128/mbio.01621-25)
Supplement: Legends — Supplemental figure legends. [file mbio.01621-25-s0005.docx]

# Supplementary

**Figure S1. *P. aeruginosa C0400* can colonize and kill *C. elegans*. (a)** *C. elegans* intestinal colonization measured as cfu/mL after 24 h growth on *E. coli* OP50 or *P. aeruginosa* strains (PA14 or C0400). Data shown as mean ± SD of three biological replicates, each in single technical replicate. Values were compared using a one-way ANOVA with a Tukey multiple comparison test with *P ≤ 0.05, **P ≤ 0.01, and ns representing not significant. **(b)** Representative survival curve of worms exposed to OP50, PA14, or C0400 in slow killing assay. Curves were compared using the Log-rank (Mantel Cox) test with one pair compared at a time, *P ≤ 0.05 and **** P ≤ 0.0001. Each condition is 150-200 animals. The colour of the significance value represents the comparison performed, with black represent comparison to control and green to PA14.

**Figure S2. Longer treatment duration shows variability in bacterial colonization.** *C. elegans* intestinal colonization measured as cfu/mL/worm after 48h C0400 infection followed by **(a-b)** 4 h **or (c-d)** 18h treatment with phage Hali (1 x 10^9^ PFU) and/or ciprofloxacin. Each graph shows a single biological replicate carried out with approximately 50-100 worms. UN denotes untreated. Data shown as mean ± SD where each data point is a technical replicate of bacterial plating.

**Figure S3.** C**iprofloxacin alone at 92 ug/mL could rescue life span of *P. aeruginosa* infected worms on standard *E. coli* OP50 diet after treatment.** Survival curve of worms on OP50 after 4 h *P. aeruginosa* infection followed by 18 h phage Hali and/or ciprofloxacin treatment, **(a)** low dose 2 µg/mL and **(b)** high dose 92 µg/mL**.** Approximately 50 worms were added to each condition at the time of treatment. Independent biological replicates are shown for each concentration. Survival curves were compared using the Log-rank (Mantel Cox) test with one pair compared at a time, ns = not significant, * P ≤ 0.05, *** P ≤ 0.001, and **** P ≤ 0.0001. Phage alone and antibiotic alone were compared to the untreated, and tPAS to the phage alone and antibiotic alone, as denoted by colour of the significance value.

**Figure S4.** **Shorter infection prior to treatment does not provide enough resolution to measure bacterial colonization.** *C. elegans* intestinal colonization measured as log10 cfu/mL/worm after 4h C0400 infection followed by 18 h treatment with phage Hali (1 x 10^9^ PFU) and/or ciprofloxacin. UN denotes untreated. Data shown as mean ± SD where each data point is a biological replicate performed in technical triplicates with $\sim$100 worms each. Values were compared using a one-way ANOVA with a multiple comparison Tukey test post-hoc. tPAS conditions were not statistically different (ns) than the UN, phage alone, and respective antibiotic alone concentration.
